# Novel LncRNA OXCT1-AS1 indicates poor prognosis and contributes to tumorigenesis by regulating miR-195/CDC25A axis in glioblastoma

**DOI:** 10.1186/s13046-021-01928-4

**Published:** 2021-04-08

**Authors:** Chen Zhong, Qian Yu, Yucong Peng, Shengjun Zhou, Zhendong Liu, Yong Deng, Leiguang Guo, Shiguang Zhao, Gao Chen

**Affiliations:** 1 Department of Neurosurgery, Second Affiliated Hospital, School of Medicine, Zhejiang University, Jiefang Road 88th, Hangzhou, 310016 Zhejiang Province People’s Republic of China; 2 Department of Pharmacology, The State-Province Key Laboratories of Biomedicine Pharmaceutics of China, College of Pharmacy of Harbin Medical University, No. 157 Baojian Street, Nangang District, Harbin, 150001 Heilongjiang Province People’s Republic of China; 3 Department of Neurosurgery, The First Affiliated Hospital of Harbin Medical University, No. 23 Youzheng Street, Nangang District, Harbin, 150001 Heilongjiang Province People’s Republic of China

**Keywords:** Glioblastoma, Competing endogenous RNA network, Long noncoding RNA, OXCT1-AS1, Proliferation

## Abstract

**Background:**

Long noncoding RNAs (lncRNAs) contribute to multiple biological processes in human glioblastoma (GBM). However, identifying a specific lncRNA target remains a challenge. In this study, bioinformatics methods and competing endogenous RNA (ceRNA) network regulatory rules were used to identify GBM-related lncRNAs and revealed that OXCT1 antisense RNA 1 (OXCT1-AS1) is a potential therapeutic target for the treatment of glioma.

**Methods:**

Based on the Gene Expression Omnibus (GEO) dataset, we identified differential lncRNAs, microRNAs and mRNAs and constructed an lncRNA-associated ceRNA network.

The novel lncRNA OXCT1-AS1 was proposed to function as a ceRNA, and its potential target miRNAs were predicted through the database LncBase Predicted v.2. The expression patterns of OXCT1-AS1 in glioma and normal tissue samples were measured. The effect of OXCT1-AS1 on glioma cells was checked using the Cell Counting Kit 8 assay, cell colony formation assay, Transwell assay and flow cytometry in vitro. The dual-luciferase activity assay was performed to investigate the potential mechanism of the ceRNA network. Finally, orthotopic mouse models of glioma were created to evaluate the influence of OXCT1-AS1 on tumour growth in vivo.

**Results:**

In this study, it was found that the expression of lncRNA OXCT1-AS1 was upregulated in both The Cancer Genome Atlas (TCGA) GBM patients and GBM tissue samples, and high expression of OXCT1-AS1 predicted a poor prognosis. Suppressing OXCT1-AS1 expression significantly decreased GBM cell proliferation and inhibited cell migration and invasion. We further investigated the potential mechanism and found that OXCT1-AS1 may act as a ceRNA of miR-195 to enhance CDC25A expression and promote glioma cell progression. Finally, knocking down OXCT1-AS1 notably attenuated the severity of glioma in vivo.

**Conclusion:**

OXCT1-AS1 inhibits glioma progression by regulating the miR-195-5p/CDC25A axis and is a specific tumour marker and a novel potential therapeutic target for glioma treatment.

**Supplementary Information:**

The online version contains supplementary material available at 10.1186/s13046-021-01928-4.

## Background

Glioma is an aggressive subtype of primary brain tumours with an extremely poor prognosis; GBM is the most severe type of glioma and accounts for approximately 1/5–1/4 of primary intracranial malignancies [1, 2]. Patients with GBM have a poor prognosis, and the 5-year survival rate is less than 10% [3, 4]. The prognosis of patients diagnosed with malignant gliomas remains dismal, although treatments such as radical surgery, radiotherapy, and chemotherapy are valuable in managing these tumours [5]. Additionally, prognostic biomarkers and therapeutic targets for gliomas have not been fully characterised [6, 7]. Therefore, it is necessary to identify novel biomarkers in glioma and reveal the molecular mechanism underlying glioma progression, to improve the early diagnosis and effective treatment of the disease.

In the eukaryotic cell genome, more than 90% of human transcripts have limited protein-coding capacity but encode noncoding RNAs, including microRNAs (miRNAs), lncRNAs, and circular RNAs (circRNAs) [8–10]. LncRNAs are noncoding RNA molecules of more than 200 nucleotides in length that are transcribed by RNA polymerase II and exert several regulatory functions at both the transcriptional and post-transcriptional levels [11]. Although lncRNAs do not encode proteins, they regulate gene expression in various ways, such as genome modification, transcriptional activation, transcriptional interference and chromosome sedimentation [12, 13]. Accumulating evidence has shown that the abnormal expression of lncRNAs is closely related to the pathogenesis, progression and prognosis of malignant tumours, including GBM [14]. Mechanistically, the ceRNA hypothesis suggests that lncRNAs function as competing endogenous RNAs in multiple human malignancies to regulate the miRNA-mRNA axis [15]. Increasing data suggest that lncRNA/miRNA/target gene axis play important roles in GBM. For example, LncRNA HOXA-AS3/miR-455-5p/USP3 axis promotes the malignancy of glioblastoma [16] and Linc00152/miR-103a-3p/FEZF1 axis promotes the malignant progression of glioma stem cells [17].

The present study aimed to better understand the pathological process of GBM at the genome level and identify new and specific lncRNA targets. We constructed an lncRNA-related ceRNA network in GBM and identified lncRNA OXCT1-AS1 as a potential specific prognostic biomarker and therapeutic target in GBM. TCGA and quantitative real-time PCR (qRT-PCR) assays revealed that the expression of OXCT1-AS1 was significantly increased in GBM tissue samples and cell lines and that patients with higher OXCT1-AS1 expression had a worse prognosis. Additionally, we demonstrated that OXCT1-AS1 promotes GBM proliferation by regulating the miR-195/CDC25A axis in GBM. Our study is the first to report the expression pattern, biological function and potential regulatory mechanism of lncRNA OXCT1-AS1 in GBM and may provide a novel diagnostic biomarker and therapeutic target for GBM.

## Materials and methods

### Data collection

Series matrix files of the GSE4290 dataset containing the mRNA microarray data of tissues samples from 23 epileptic and 81 GBM patients [18], the GSE90603 dataset containing the miRNA microarray data of 16 fresh-frozen GBM multiforme and 7 healthy brain tissue samples, and the GSE104267 dataset containing the mRNA microarray data of 9 tumour and 3 healthy tissue samples were downloaded from the GEO database (https://www.ncbi.nlm.nih.gov/geo/) [19]. All 26 glioma specimens and paired non-tumour tissues used in this study were collected from glioma patients who had undergone surgery. All the GBM samples were immediately frozen in liquid nitrogen until RNA was extracted.

### Patient tissue preparation

A total of 26 pairs of GBM samples and their adjacent normal tissues were obtained from the First Affiliated Hospital of Harbin Medical University between 2018 and 2020. For qRT-PCR and western blot analysis, tissues were immediately frozen in liquid nitrogen. All experimental ethics were approved by the Institutional Animal Care and Use Committee at Harbin Medical University (No. HMUIRB-2008-06).

### ceRNA network construction

The differentially expressed mRNAs (DEmRNAs), differentially expressed lncRNAs (DElncRNAs), and differentially expressed miRNAs (DEmiRNAs) in the GSE4290, GSE90603 and GSE104267 datasets were identified using the limma package in R [20]. Differential expression analyses were performed using the limma settings of an FDR threshold of 0.05 and an FC lower threshold of |log(FC)| ≥ 1. We used online tools (http://bioinfogp.cnb.csic.es/tools/venny/) to conduct integrated bioinformatics analyses [21, 22]. The miRcode database (http://www.mircode.org) was used to define the relationships between the DElncRNAs and DEmiRNAs. Target genes were predicted by miRTarBase, TargetScan, and miRDB. The DElncRNA-DEmiRNA-DEmRNA network was constructed using Cytoscape (version 3.6.1) [23].

### Functional annotation and pathway analyses

We conducted Gene Ontology (GO) enrichment and Kyoto Encyclopedia of Genes and Genome (KEGG) pathway analyses for the mRNAs involved in the constructed lncRNA-related network using the R package “clusterprofiler”, with a set of cut-off criteria at *P* < 0.05 [24]. The online database Retrieval of Interacting Gene (STRING) was used to construct a protein-protein interaction (PPI) network [25]. DEmRNAs were incorporated into the PPI network when they had a combined score greater than 0.4 [26].

### Cell lines and cell culture

All human GBM cell lines (A172, LN229, U87, U251, and U373) and a normal human astrocyte cell line (NHA) were obtained from the China Infrastructure of Cell Line Resource. The cells were cultured and preserved in DMEM (GIBCO-BRL, 11965092) supplemented with 10% FBS, 100 U/mL penicillin and 100 mg/mL streptomycin in humidified air at 37 °C with 5% CO_2_.

### Cell transfection

OXCT1-AS1 inhibitor shRNA and an empty vector were commercially synthesised by General Biology (Anhui, China). The plasmids were transfected at 2.5 μg/well in a 6-well plate. All the transfections were performed using Lipofectamine 2000 (Invitrogen, Carlsbad, USA, 11668019). The transfection efficiency was assessed by qRT-PCR analysis. Subsequent experiments were performed at 48 h post transfection.

### qRT-PCR analysis

Total RNA was extracted from glioma tissues and cell lines using TRIzol reagent (Ambion Life Technologies, USA, A33254). Reverse transcription was performed using a First Strand cDNA Synthesis Kit (TOYOBO Life Science, Shanghai, China, FSQ-101). Next, qRT-PCR analyses were performed using the Universal SYBR-Green Master Mix (Roche, Germany, 04707516001). GAPDH was used as an endogenous control for lncRNAs and mRNAs. U6 was used as endogenous controls for miRNAs. The 2^− ▵▵C*t*^ method was used to analyse the results.

### Cell proliferation assay

The transfected cells were cultured in 96-well plates at 1 × 10^4^ per well and incubated for 24, 48, and 72 h. Cell proliferation was assayed using the Cell Counting Kit 8 assay (CCK-8; MedChem Express, China, HY-K0301) according to the manufacturer’s protocol.

### Colony formation assay

The cells were plated at a density of 5 × 10^3^ cells/well in 96-well plates. After 24 h of transfection with sh-OXCT1-AS1 and the empty vector, the cells were seeded into six-well plates (500 cells per well) and cultured for 2 weeks. Next, 0.1% crystal violet was used to stain clones, and cells were photographed using a ChemiDoc™ MP system (Bio-Rad, USA). The number of colonies was counted using ImageJ.

### Immunofluorescence staining

After transfection, the cells were fixed with 4% paraformaldehyde, permeabilised with Triton X-100 and blocked with 5% bovine serum albumin (BSA; BOSTER, USA, AR0004). Next, the cells were incubated with the Ki-67 primary antibody overnight at 4 °C, followed by Alexa Fluor 594 AffiniPure goat anti-rabbit IgG secondary antibody (ZSGB-BIO, China, ZF-0516) and DAPI (Beyotime, China, C1005). The results were determined by fluorescence microscopy the next day.

### Flow cytometry analysis of the cell cycle

After transfection, the cells were harvested and stained using the Cycletest™ PLUS DNA Reagent Kit (BD Biosciences, 340242). Next, the samples were analysed using an Accuri C5 flow cytometer (BD Biosciences) to determine the distribution of the cells in the G0-G1, S, and G2-M phases.

### Western blotting

Total cell protein was lysed using RIPA buffer (Beyotime Institute of Biotechnology, Beijing, China, P0013B) containing protease and phosphatase inhibitors. Equal amounts of lysates were separated on 12.5% SDS-PAGE gels and transferred to PVDF membranes (Millipore, Billerica, MA). After blocking in 5% skim milk with TBST, the membranes were incubated with primary antibodies overnight at 4 °C. The membranes were incubated with secondary antibodies at room temperature for 1 h. The protein bands were visualised by ChemiDoc™ MP System.

### Transwell assay

Eight-micrometre Transwell chambers (Corning Company, NY) were used to conduct migration assays. After transfection, the cells were planted into the upper chambers (1 × 10^5^ cells/well) and cultured for 24 h. The chamber was fixed with 4% paraformaldehyde and stained with 0.1% crystal violet for 20 ~ 30 min. After washing out the crystal violet, the stained cells were counted under a microscope. Next, 100 μl of Matrigel (BD Biosciences, 354230) was added to the upper chambers, and the assay was performed as previously described to detect cell invasion ability.

### Luciferase reporter assay

Fragments of OXCT1-AS1 and CDC25A 3’UTR containing miR-195 binding sites were amplified and cloned into the psiCheck2 reporter vector (Promega, Shanghai, China). Next, HEK293 cells were cotransfected with reporter vector and miR-195 mimics for 48 h. Luciferase activity was measured using the Dual Luciferase Reporter Assay (Promega, E1910), normalising firefly (experimental group) luciferase to Renilla (control group) activity.

### In vivo tumour formation assay

Four-week-old BALB/C nude mice (15–20 g) were obtained from the Vital River Animal Center (Beijing, China) and randomly divided into two groups. Mice anaesthetised with isoflurane were placed in a stereotaxic frame, and the skull was exposed for intracranial injections or infusions. After stable transfection with the empty vector or shRNA, LN229 cells were collected, and 5 × 10^6^ resuspended cells were used for each intracranial injection. Magnetic resonance imaging was performed to evaluate the intracranial lesions every 15 days after the vaccinations. The tumour volume was assessed and calculated (volume = 4/3π(a*b*c), a,b,c are the 1/2 maximum diameter at coronal, sagittal and axial position) every 3 days. The survival time of the mice was recorded, and Kaplan-Meier survival curves were plotted for each group.

### Immunohistochemistry

Formalin-fixed tumour tissues were embedded in paraffin and sliced into 5-μm-thick sections. After blocking with 5% BSA, the sections were incubated with the CDC25A primary antibody (1:500; Affinity Biosciences, AF6252) at 4 °C overnight and secondary antibodies at 37 °C for 30 min. Next, the samples were visualised using the diaminobenzidine substrate kit (Abcam, ab64238) for 10 min. After intensive washing, the samples were counterstained with haematoxylin, dehydrated and covered with a coverslip.

### Statistical analysis

The data from three independent experiments were shown as means ± standard deviations (SD). Two-group statistical analysis was performed using Student’s t test (two-tailed). For multiple group statistical analysis, we used one-way ANOVA with SPSS 19.0 (IBM, USA). A *P*-value< 0.05 was considered statistically significant.

## Results

### Construction of the ceRNA network in GBM

**Fig. 1:**
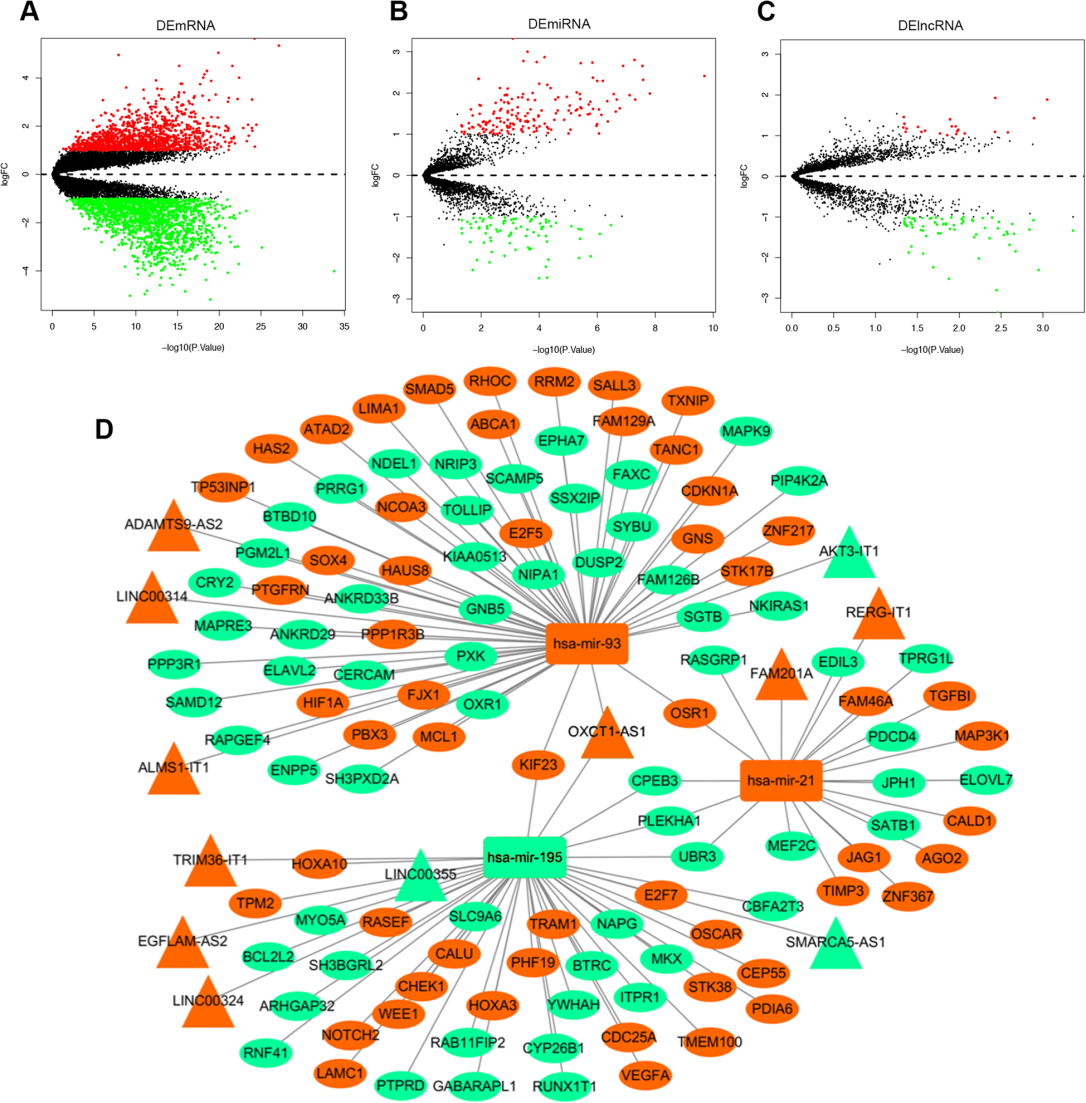
Expression distribution of differential lncRNAs, miRNAs and mRNAs. **a** Differentially expressed mRNAs from GSE4290. **b** Differentially expressed miRNAs from GSE104267. **c** Differentially expressed mRNAs from GSE90603. The red plots represent the upregulated targets with statistical significance, and the green plots represent the downregulated targets with statistical significance. **d** ceRNA network in GBM. The red nodes represent upregulated differentially expressed RNAs, and the green nodes represent downregulated differentially expressed RNAs. DElncRNAs, DEmiRNAs, and DEmRNAs are represented by triangles, rectangles, and ellipses, respectively

From the GEO database, we downloaded the microarray data of GSE4290, which included tissue samples from 23 epileptic and 81 GBM patients, and the microarray data of GSE90603, which included 16 fresh-frozen GBM multiforme and 7 healthy brain tissue samples, and the microarray data of GSE104267, including 9 GBM and 3 epileptic tissue samples. We identified 1292 upregulated mRNAs and 1709 downregulated mRNAs in GSE4290 (Fig. 1a, S1A), 170 upregulated miRNAs and 81 downregulated miRNAs (Fig. 1b, S1B) in GSE90603, and 21 upregulated lncRNAs and 69 downregulated lncRNAs (Fig. 1c, S1C) in GSE104267, with an FC lower threshold of |log (FC)| ≥ 1 and *P* < 0.05. We selected the interaction pairs of lncRNAs and miRNAs based on the miRcode database and interaction pairs of miRNAs and mRNAs based on the miRDB database, TargetScan and miRTarBase. We constructed an lncRNA-miRNA-mRNA competing network. Finally, 12 DElncRNAs, 3 DEmiRNAs, and 116 DEmRNAs were employed to construct the ceRNA network using Cytoscape (Fig. 1d). We found that lncRNA OXCT1-AS1 has a connection with two of the three DEmiRNAs; therefore, we focused on OXCT1-AS1 in the subsequent experiments. According to the ceRNA network hypothesis, we found that OXCT1-AS1 expression was only inversely related to the miR-195 level. Hence, we hypothesised that miR-195 might be the downstream target of OXCT1-AS1 in the ceRNA network.

### GO functional annotation and KEGG pathway enrichment analyses

**Fig. 2:**
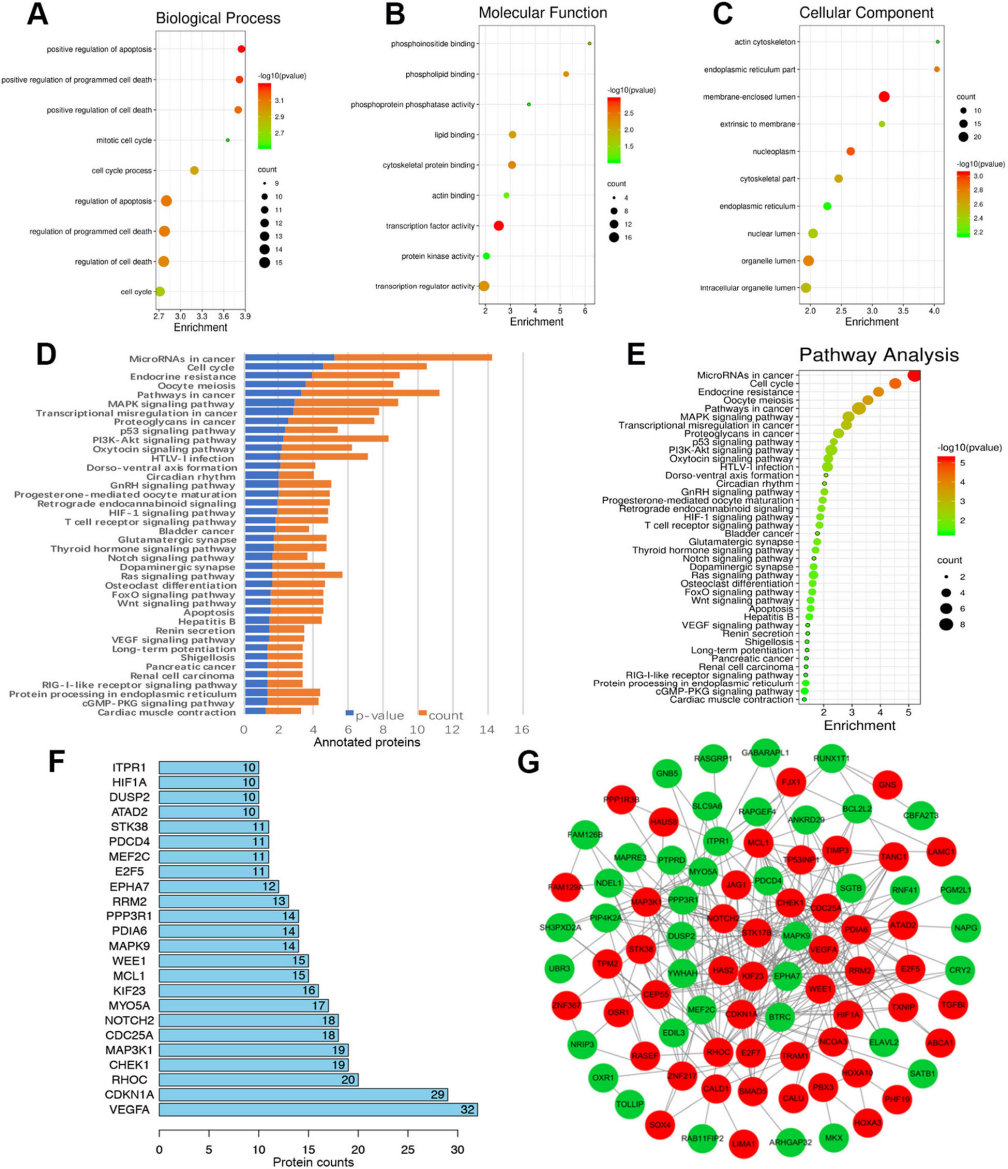
GO functional annotation and KEGG pathway enrichment analyses and hub genes involved in these pathways. **a** GO functional enrichment in cell biological processes. **b** GO functional enrichment in cell molecular function. **c** GO functional enrichment in cellular function. **d**, **e** KEGG cell signalling pathway enrichment of the ceRNA network. **f** Hub genes with a high degree of connectivity among DEmRNAs. **g** PPI network of DEmRNAs obtained from the ceRNA network. The red nodes represent upregulated DEmRNAs, and the green nodes represent downregulated DEmRNAs. PPI, protein-protein interaction

We Used the ClusterProfile package in R software to perform the GO functional enrichment and the KEGG pathway enrichment analyses. GO analysis showed that the DEmRNAs we obtained were associated with “cell cycle,” “membrane enclosed lumen,” “transcription factor activity,” and so on (Fig. 2a-d, Table S1, S2 and S3). KEGG pathway analysis showed that the DEmRNAs were enriched in cancer associated pathways, such as MicroRNA in cancer, cell cycle and so on (Fig. 2f, Table 1). Both GO and KEGG analysis all enriched in cell cycle process. From the ceRNA network, we searched the cell cycle related DEmRNAs can be regulated by miR-195 and found cell division cycle 25A (CDC25A) was the only candidate. We also used the STRING database to explore the hub genes from the DEmRNAs and constructed the PPI network (Fig. 2g). We identified the top 10 DEmRNAs as the hub genes based on their linkage degree, including CDC25A (Fig. 2f). Thus, CDC25A might be the component of OXCT1-AS1/miR-195 axis regulated ceRNA network.

**Table 1:** KEGG cell signaling pathway analysis of DEmRNAs in lncRNA related ceRNA networks

ID	Description	Adjusted ***P*** values	Counts	Gene names
hsa05206	MicroRNAs in cancer	0.00000602	9	MCL1/CDC25A/NOTCH2/BCL2L2/VEGFA/CDKN1A/KIF23/SOX4/TIMP3
hsa04110	Cell cycle	0.0000297	6	CDC25A/WEE1/CHEK1/CDKN1A/E2F5/YWHAH
hsa01522	Endocrine resistance	0.00011607	5	NCOA3/CDKN1A/MAPK9/JAG1/NOTCH2
hsa04114	Oocyte meiosis	0.00027439	5	PPP3R1/BTRC/ITPR1/YWHAH/CPEB3
hsa05200	Pathways in cancer	0.00056634	8	RASGRP1/RUNX1T1/VEGFA/CDKN1A/GNB5/HIF1A/MAPK9/LAMC1
hsa04010	MAPK signaling pathway	0.00132625	6	PPP3R1/RASGRP1/DUSP2/MAP3K1/MAPK9/MEF2C
hsa05202	Transcriptional misregulation in cancer	0.00157405	5	CDKN1A/RUNX1T1/MEF2C/HOXA10/PBX3
hsa05205	Proteoglycans in cancer	0.0029711	5	CDKN1A/ITPR1/TIMP3/VEGFA/HIF1A
hsa04115	p53 signaling pathway	0.00438531	3	CDKN1A/CHEK1/RRM2
hsa04151	PI3K-Akt signaling pathway	0.00536491	6	VEGFA/MCL1/CDKN1A/GNB5/YWHAH/LAMC1
hsa04921	Oxytocin signaling pathway	0.00681985	4	CDKN1A/PPP3R1/ITPR1/MEF2C
hsa05166	HTLV-I infection	0.00730939	5	CDKN1A/PPP3R1/MAP3K1/CHEK1/TP53INP1
hsa04320	Dorso-ventral axis formation	0.00825292	2	CPEB3/NOTCH2
hsa04710	Circadian rhythm	0.00935463	2	BTRC/CRY2
hsa04912	GnRH signaling pathway	0.00943716	3	MAP3K1/ITPR1/MAPK9
hsa04914	Progesterone mediated oocyte maturation	0.0108457	3	MAPK9/CPEB3/CDC25A
hsa04723	Retrograde endocannabinoid signaling	0.01205547	3	ITPR1/MAPK9/GNB5
hsa04066	HIF-1 signaling pathway	0.01333979	3	CDKN1A/HIF1A/VEGFA
hsa04660	T cell receptor signaling pathway	0.01401011	3	PPP3R1/RASGRP1/MAPK9
hsa05219	Bladder cancer	0.01648587	2	CDKN1A/VEGFA
hsa04724	Glutamatergic synapse	0.01688062	3	PPP3R1/ITPR1/GNB5
hsa04919	Thyroid hormone signaling pathway	0.01923358	3	NCOA3/HIF1A/NOTCH2
hsa04330	Notch signaling pathway	0.02190805	2	JAG1/NOTCH2
hsa04728	Dopaminergic synapse	0.0226392	3	ITPR1/MAPK9/GNB5
hsa04014	Ras signaling pathway	0.0227743	4	RASGRP1/VEGFA/MAPK9/GNB5
hsa04380	Osteoclast differentiation	0.02445721	3	PPP3R1/OSCAR/MAPK9
hsa04068	FoxO signaling pathway	0.02539501	3	CDKN1A/GABARAPL1/MAPK9
hsa04210	Apoptosis	0.02882821	3	MAPK9/ITPR1/MCL1
hsa04310	Wnt signaling pathway	0.02882821	3	PPP3R1/BTRC/MAPK9
hsa05161	Hepatitis B	0.03195714	3	CDKN1A/MAP3K1/MAPK9
hsa04370	VEGF signaling pathway	0.03660229	2	PPP3R1/VEGFA
hsa04924	Renin secretion	0.03660229	2	PPP3R1/ITPR1
hsa05131	Shigellosis	0.03864514	2	BTRC/MAPK9
hsa04720	Long-term potentiation	0.03864514	2	PPP3R1/ITPR1
hsa05212	Pancreatic cancer	0.04073055	2	VEGFA/MAPK9
hsa05211	Renal cell carcinoma	0.04178893	2	HIF1A/VEGFA
hsa04622	RIG-I-like receptor signaling pathway	0.0428576	2	MAP3K1/MAPK9
hsa04141	Protein processing in endoplasmic reticulum	0.0429848	3	MAPK9/TRAM1/PDIA6
hsa04022	cGMP-PKG signaling pathway	0.04681452	3	PPP3R1/ITPR1/MEF2C
hsa04260	Cardiac muscle contraction	0.04835144	2	TPM2/SLC9A6

### LncRNA OXCT1-AS1 is highly expressed in GBM and associated with prognosis

**Fig. 3:**
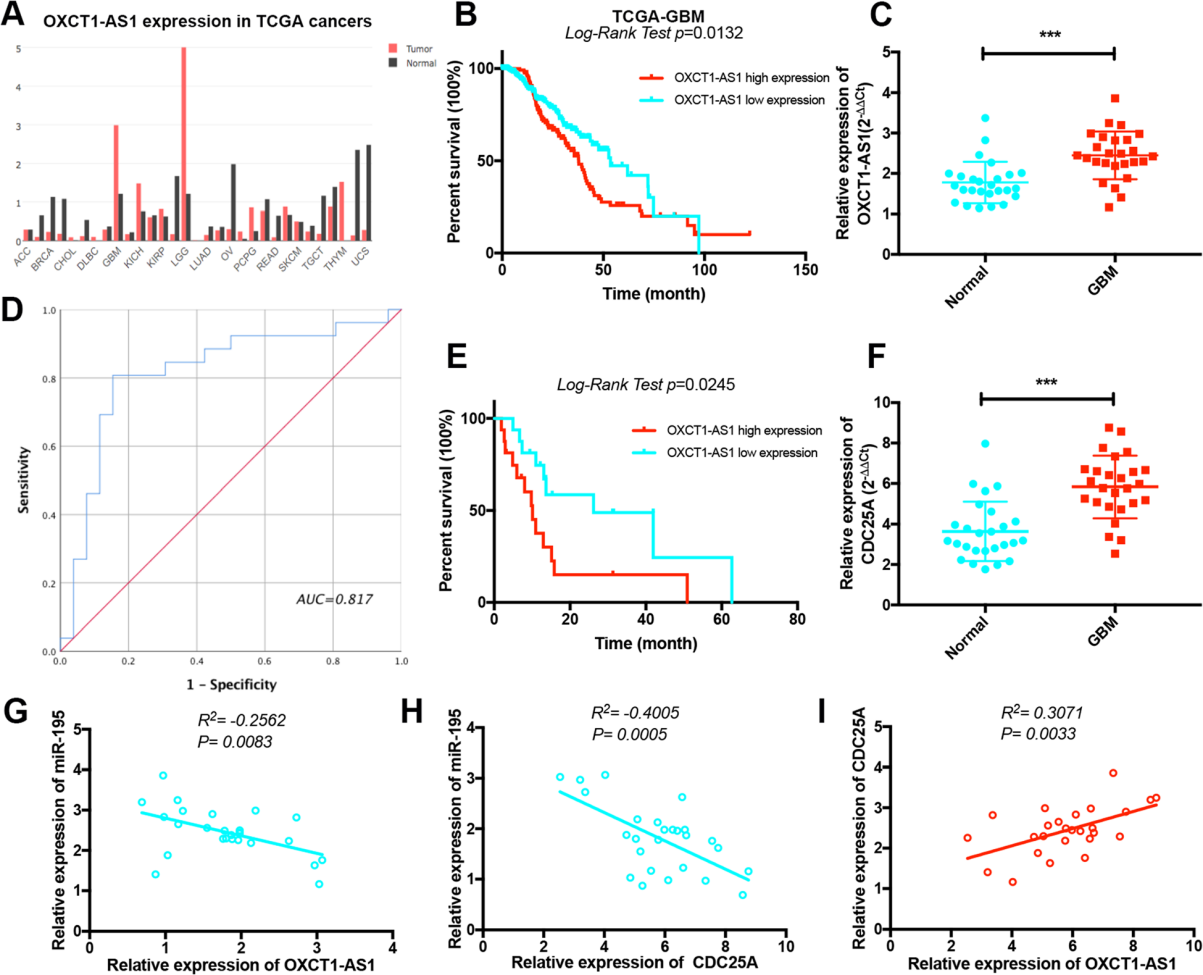
LncRNA OXCT1-AS1 is highly expressed in GBM and associated with prognosis. **a** OXCT1-AS1 expression patterns in different types of solid cancers from the TCGA database. **b** Kaplan-Meier curve analysis of OXCT1-AS1 based on TCGA GBM samples. **c** The expression of OXCT1-AS1 was examined by qRT-PCR in GBM tissues compared with that in corresponding adjacent normal tissues. **d** The ROC curve was constructed using SPSS. The area under the curve was 0.817. The sensitivity and specificity were 0.769 and 0.808, respectively. **e** The survival time of GBM patients was longer in the OXCT1-AS1 low expression group than in the high expression group. **f** The expression of CDC25A was examined by qRT-PCR in GBM tissues compared with that in corresponding adjacent normal tissues. GAPDH was used to normalize the gene expression. **g** The negative expression correlation between OXCT1-AS1 and miR-195 in GBM tissues was analysed. **h** Correlation between the expression of miR-195 and CDC25A. **i** Positive expression correlation between OXCT1-AS1 and CDC25A. The statistical bars represent SD (*p < 0.05, **p < 0.01, and ***p < 0.001)

We first examined the expression pattern of lncRNA OXCT1-AS1 in various common solid cancers based TCGA database analysis and found that OXCT1-AS1 was aberrantly expressed in GBM and low-grade glioma (LGG) (Fig. 3a). In GBM, patients with high OXCT1-AS1 expression had a lower median survival (37.75 months) than those with low OXCT1-AS1 expression (53.52 months). The median survival status significantly differed between the high OXCT1-AS1 and low OXCT1-AS1 expression groups (HR = 1.52; 95% CI = 1.094–2.163; *P* value = 0.0132; Fig. 3b). We also analysed 26 pairs of GBM samples and their adjacent normal tissues, and the expression of OXCT1-AS1 was remarkably increased in the GBM samples (Fig. 3c). Kaplan-Meier analysis was used to evaluate OXCT1-AS1-related patient survival and revealed that higher OXCT1-AS1 expression was associated with poor survival (HR = 1.922; 95% CI = 1.154–3.203; *P* value = 0.0245; Fig. 3e). We wondered whether OXCT1-AS1 could be an indicator for the diagnosis of GBM. We used the 26 paired adjacent normal tissues as controls to build the ROC curve (Fig. 3d). The sensitivity and specificity were 0.769 and 0.808, respectively, and the cut-off value was 1.1545. The area under the curve was 0.817 (95% CI = 0.692–0.941; *P* < 0.000), and the Youden index was 0.577. In the univariable and multivariable Cox regression models, we found that OXCT1-AS1 expression was an indicator of GBM (HR = 0.468; 95% CI = 0.094–0.361; *P* value = 0.014; Table 2). qRT-PCR assays also demonstrated that CDC25A was upregulated in glioma tissues (Fig. 3f), in contrast to miR-195 expression but consistent with OXCT1-AS1 expression (Fig. 3g-i).

**Table 2:** Univariable and multivariable Cox regression analysis in GBM

Characteristics	Subset	Univariate analysis Hazard ratio (95%CI)	*P* value	Multivariate analysis Hazard ratio (95%CI)	*P* value
Age	≤48/> 48	1.378 (0.451–4.214)	0.751	1.794 (0.243–4.241)	0.981
Gender	Male/Female	1.556 (0.643–3.749)	0.327	3.165 (0.981–10.210)	0.885
Chr7 gain/Chr10 loss	Yes/No	0.318 (0.126–0.804)	0.015*	0.908 (0.246–3.358)	0.054
1p/19q status	Code/Noncoded	9.036 (7.841–37.803)	0.000*	4.355 (1.121–16.443)	0.002*
IDH status	Mutation/Wild	0.630 (2.701–3.242)	0.003*	0.170 (0.042–0.691)	0.013*
MGMT promoter status	Mutation/Wild	7.151 (3.606–81.581)	0.011*	5.706 (1.986–34.619)	0.064
OXCT1-AS1	High/Low	0.701 (0.191–0.541)	0.000*	0.468 (0.094–0.361)	0.014*

In both univariable and multivariable Cox regression analyses, all characteristics were evaluated as continuous variables. *P* < 0.05 was considered statistically significant in all analyses.

### LncRNA OXCT1-AS1 has a higher expression level in GBM cell lines, and OXCT1-AS1 suppression arrests GBM cell proliferation

**Fig. 4:**
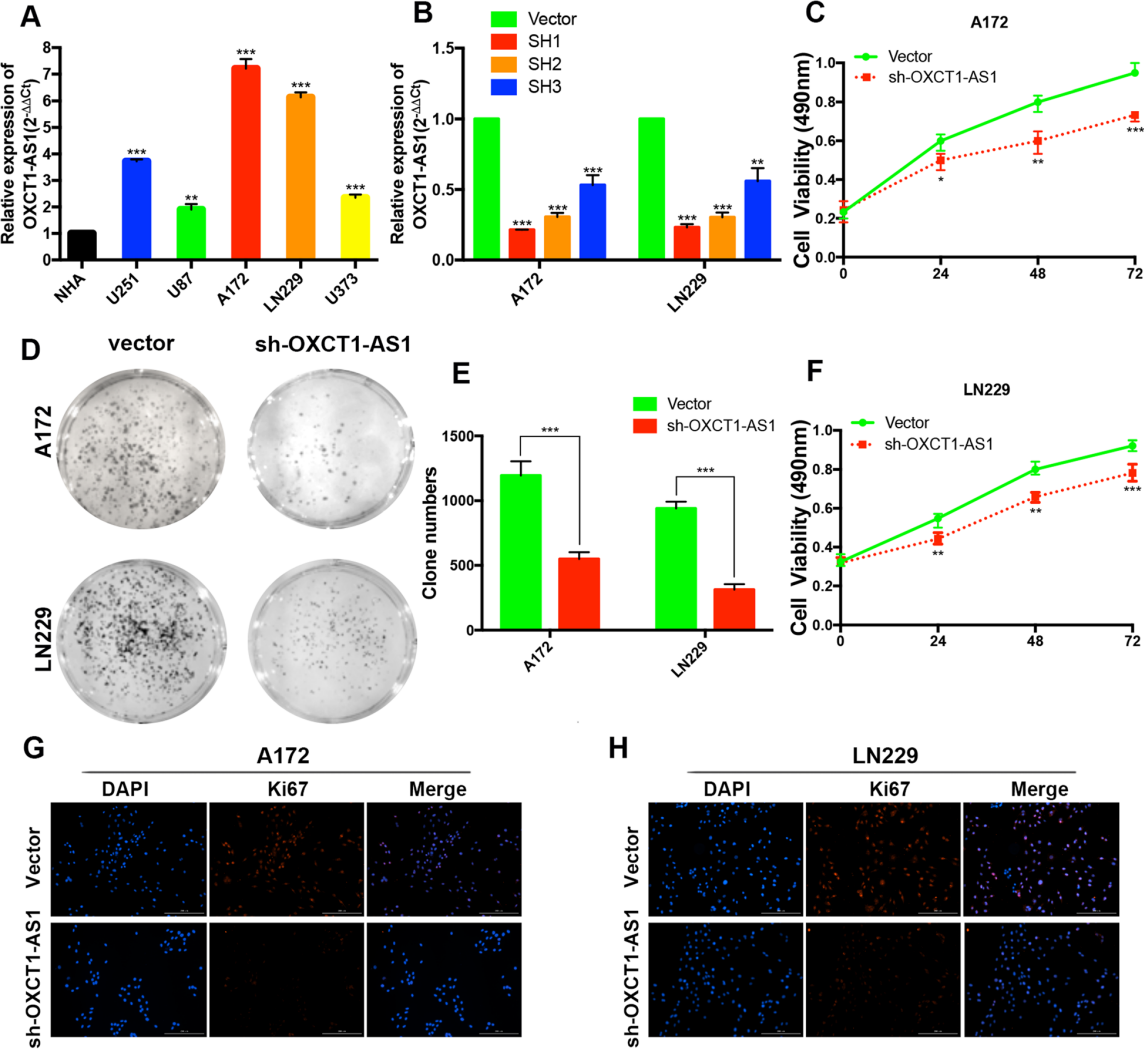
Knocking down OXCT1-AS1 markedly suppresses the proliferation of GBM cells in vivo. **a** OXCT1-AS1 expression levels in GBM cell lines compared with those in the human brain astrocyte cell line NHA. **b** Three shRNAs targeting OXCT1-AS1 and the empty vector were transfected into A172 and LN229 cells. The inhibitory efficiency was measured by qRT-PCR. **c**, **f** After transfecting sh-OXCT1-AS1 and vector, CCK-8 cell proliferation assays were performed at 0, 24, 48, and 72 h. **d**, **e** Colony formation assays performed using A172 and LN229 cells transfected with sh-OXCT1-AS1 and the empty vector. **g**, **h** Fluorescence microscopy of Ki67 expression after silencing OXCT1-AS1 or transfection with the empty vector. The scale bar represents 100 μm. The bars represent SD (*p < 0.05, **p < 0.01, ***p < 0.001)

We performed qRT-PCR assays to assess the OXCT1-AS1 expression level in GBM cell lines, and the expression levels of OXCT1-AS1 were upregulated in GBM cells compared with those in NHA cells (Fig. 4a). Because A172 and LN229 have the highest expression of OXCT1-AS1 among the GBM cell lines, we selected them for this study. We transfected shRNA into A172 and LN229 cells to suppress OXCT1-AS1 expression. Forty-eight hours post-transfection, qRT-PCR analysis was performed, and the 1st and 2nd shRNAs significantly downregulated OXCT1-AS1 expression (Fig. 4b). We selected the 1st shRNA for further experiments. The CCK-8 assay results showed that the viability of A172 and LN229 cells was obviously decreased by shRNA transfection (Fig. 4c, f). Similarly, reduced OXCT1-AS1 expression impaired the colony formation capacities of GBM cells (Fig. 4d, e). Finally, we used Ki-67 staining to measure cell proliferation. The proliferative marker Ki67 was decreased after OXCT1-AS1 knockdown (Fig. 4g, h).

### LncRNA OXCT1-AS1 promotes the migration and invasion of GBM cells in vitro

**Fig. 5:**
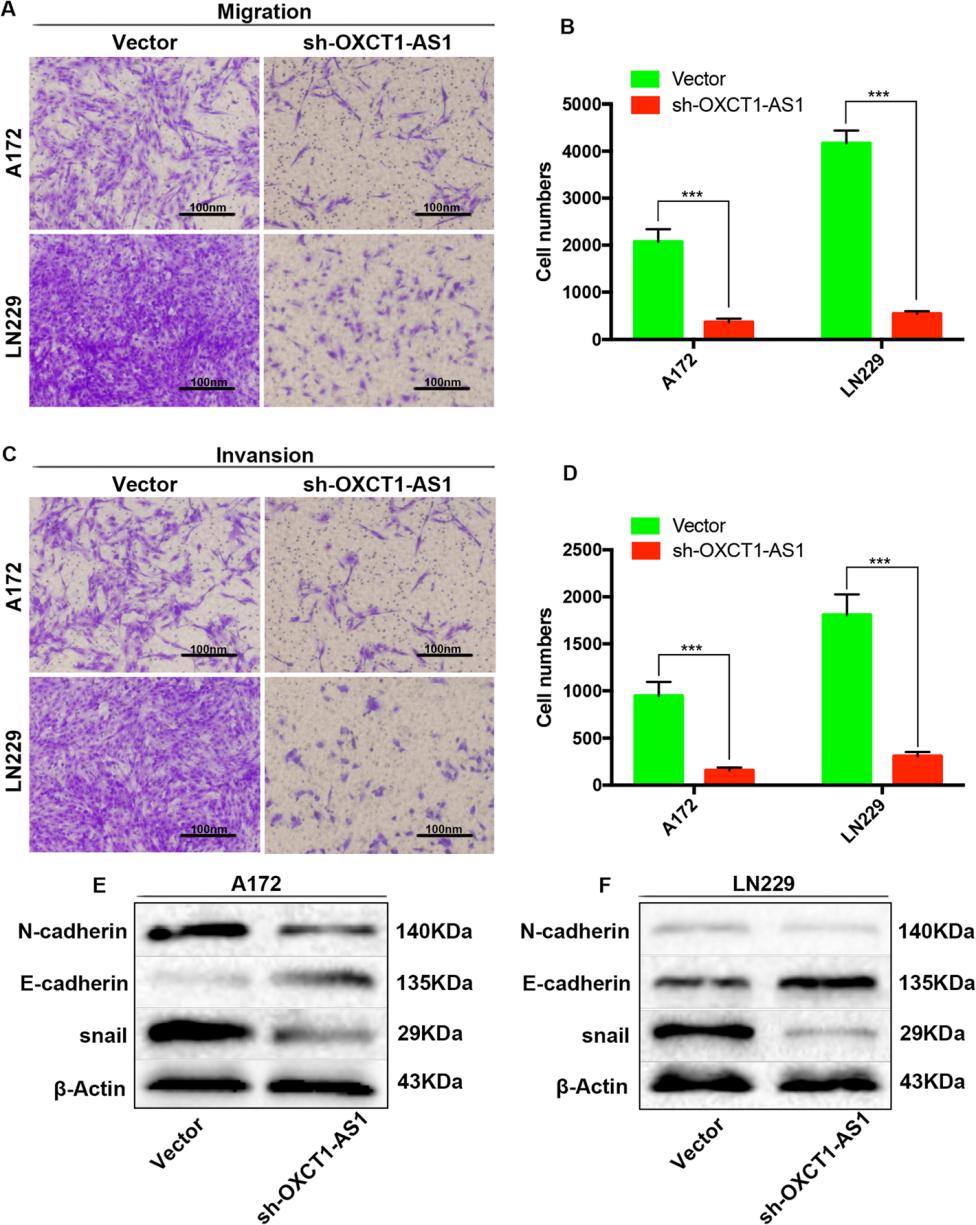
OXCT1-AS1 promotes the migration and invasion of GBM cells in vitro. **a**, **b** Transwell assays were performed to detect the migration ability of A172 and LN229 cell lines transfected with sh-OXCT1-AS1 and the empty vector. **c**, **d** The invasive ability was measured by Transwell assay with Matrigel in A172 and LN229 cell lines transfected with sh-OXCT1-AS1 or the empty vector. (**e**, **f**) After transfection with sh-OXCT1-AS1 and the empty vector, the migration- and invasion -related proteins as N-cadherin, E-cadherin and snail were examined by western blotting assay. β-Actin was used as the internal control

We also studied whether OXCT1-AS1 affected the migration and invasion of GBM cells. Cell migration and invasion potential were measured by Transwell assays. Both the migration and invasion of cells were significantly decreased after silencing OXCT1-AS1 (Fig. 5a-d). Additionally, knocking down OXCT1-AS1 increased the protein level of E-cadherin while decreasing the protein levels of N-cadherin and snail, further confirming our Transwell results (Fig. 5e and f).

### Reduced OXCT1-AS1 levels induce cell cycle arrest in GBM cells

**Fig. 6:**
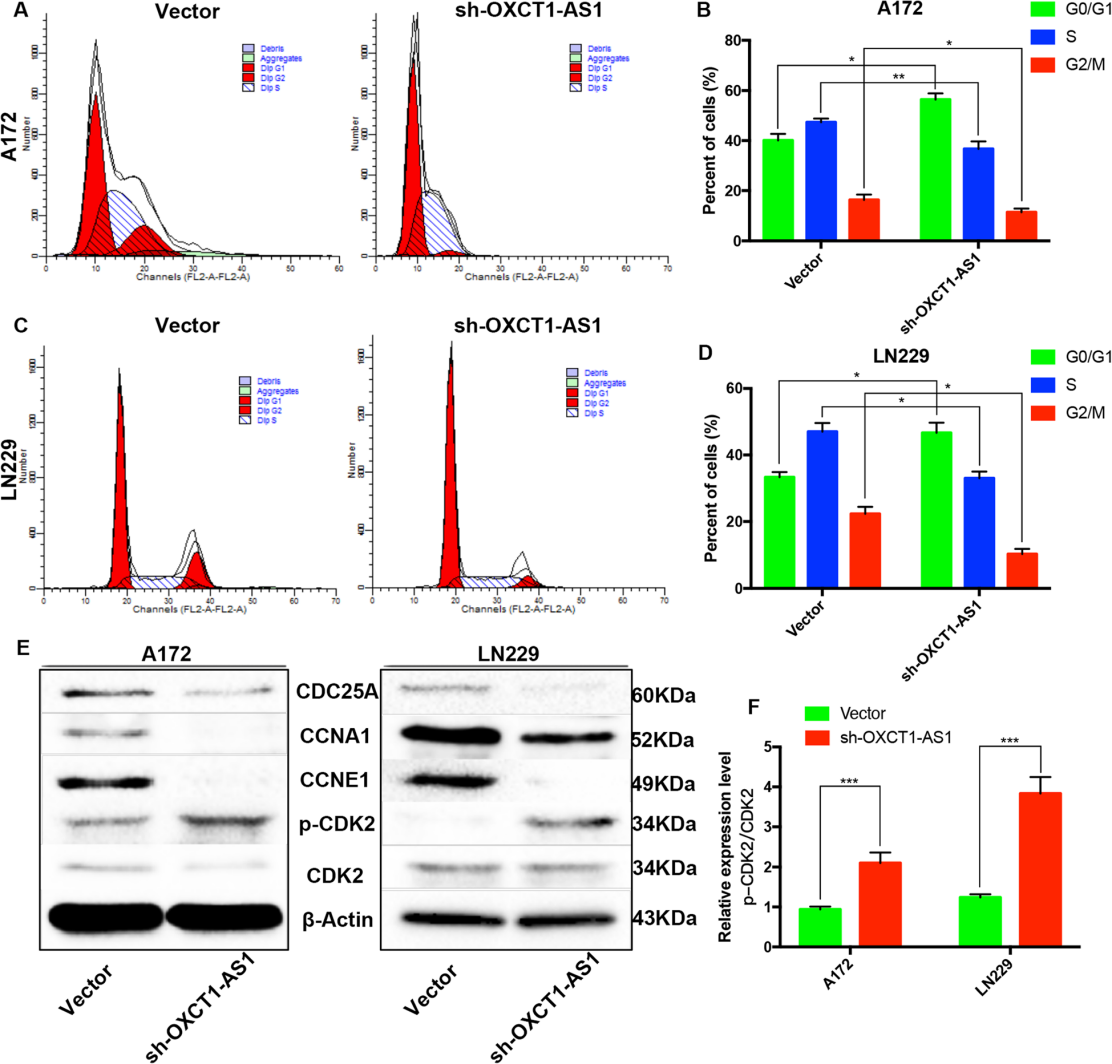
Knocking down OXCT1-AS1 suppresses CDC25A, reduces CDK2 phosphorylation and induces cell cycle arrest. **a**-**d** Flow cytometry assays were performed to analyse the cell cycle progression of the A172 and LN229 cell lines transfected with sh-OXCT1-AS1 or the empty vector. The cells in the different cell cycle phases were assessed and are shown in the bar chart. **e** Expression of cell cycle-related proteins, such as CDC25A, CDK2, CCNA1 and CCNE1, in OXCT1-AS1 knockdown cells. **f** Using total CDK2 as the internal control, the expression levels of phosphorylated CDK2 were calculated. The bars represent SD (*p < 0.05, **p < 0.01, ***p < 0.001)

Our KEGG pathway enrichment analyses indicated that the obtained hub genes may regulate cell cycle processes. Therefore, we conducted flow cytometry assays to evaluate the impact of OXCT1-AS1 on the GBM cell cycle. After silencing OXCT1-AS1 in GBM cells, the number of cells in the G0/G1 phase was increased and the number of cells in the G2/M phase was decreased (Fig. 6a-d), confirming that OXCT1-AS1 promotes GBM malignant progression, partly by regulating the cell cycle process. Additionally, by constructing a ceRNA network, we found that OXCT1-AS1 may influence the GBM cell cycle by regulating CDC25A. Furthermore, we measured the levels of proteins associated with the cell cycle, such as CDC25A, CCNA1, CCNE1 and CDK2 (the downstream tyrosine dephosphorylating target of CDC25A). Knockdown of OXCT1-AS1 significantly decreased the protein levels of CDC25A, CCNA1, and CCNE1 (Fig. 5e) and upregulated CDK2 phosphorylation levels (Fig. 5f). All these experiments further supported our hypothesis.

### OXCT1-AS1 enhances the expression level of CDC25A by competitively binding miR-195 in GBM cells

**Fig. 7:**
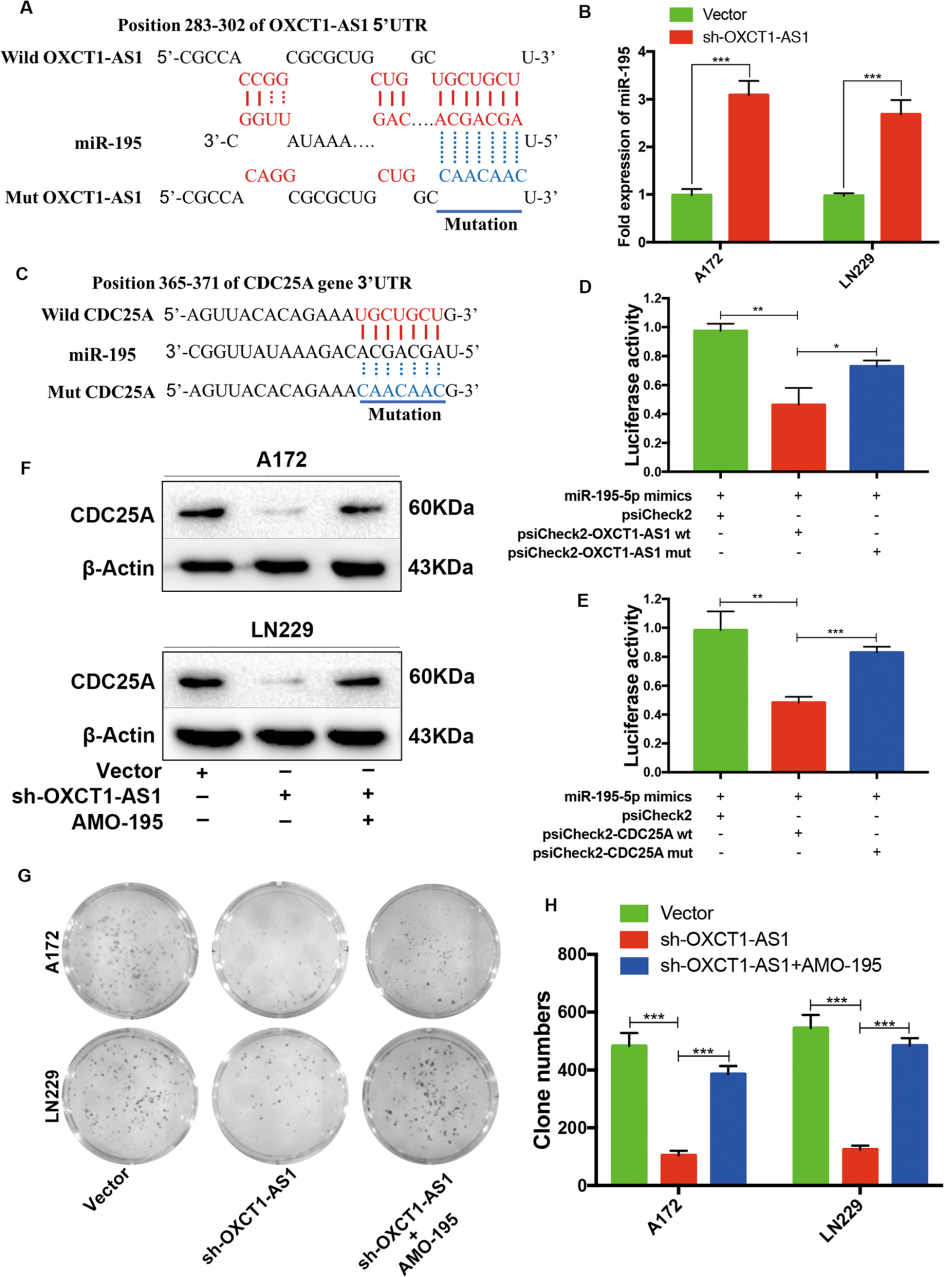
OXCT1-AS1 functions as a ceRNA in GBM by sponging miR-195 and releasing downstream CDC25A. **a** Binding sequence between OXCT1-AS1 and miR-195. **b** The expression levels of miR-195 were examined by qRT-PCR in response to OXCT1-AS1 knockdown. **c** Binding sequence between miR-195 and CDC25A. **d** The luciferase assay was used to verify the binding between OXCT1-AS1 and miR-195. **e** The luciferase assay was used to verify the binding between miR-195 and CDC25A. **f** Western blotting confirmed that the antagomir-195 (AMO-195) reversed OXCT1-AS1 knockdown-induced CDC25A suppression. **g**, **h** Colony formation assays also showed that AMO-195 partly reversed OXCT1-AS1 knockdown-induced cell proliferation arrest. The bars represent SD (*p < 0.05, **p < 0.01, ***p < 0.001)

Initially, we conducted microarray analysis and constructed a ceRNA network to explore the underlying molecular mechanism of OXCT1-AS1. To examine whether OXCT1-AS1 functions as a ceRNA in GBM cells, we used the online software LncBase V2.0 (http://carolina.imis.athena-innovation.gr) to predict the binding sites of OXCT1-AS1 and miR-195 (Fig. 7a). qRT-PCR showed that knocking down OXCT1-AS1 increased the level of miR-195 in GBM cells (Fig. 7b). To confirm the competing sponging mechanism, we constructed wild-type and mutant (mut) OXCT1-AS1 luciferase reporters separately using the psiCheck2 vector. The dual luciferase reporter assay revealed that miR-195 mimics reduced the luciferase activity of the wild-type OXCT1-AS1 reporter, but no significant change was observed in the OXCT1-AS1 mutant reporter (Fig. 7d). These results demonstrated that OXCT1-AS1 directly targeted miR-195 and that the expression of the latter was inhibited. Based on our previous microarray analysis results, CDC25A was predicted to function downstream of miR-195 in the ceRNA network. The binding sequence between miR-195 and CDC25A was predicted and is shown in Fig. 7c. Similarly, the luciferase activity of wild-type CDC25A but not mutant-type CDC25A was reduced by miR-195 mimics (Fig. 7e). We also conducted western blotting, which revealed that CDC25A expression was decreased in the OXCT1-AS1 knockdown group compared with that in the vector group, whereas the inhibitory effect could be partially reversed by adding the miR-195 inhibitor AMO-195 (Fig. 7f). Functional colony formation assays also revealed that the miR-195 inhibitor partly reversed OXCT1-AS1 knockdown-induced GBM cell growth arrest (Fig. 7g). In summary, OXCT1-AS1 promotes GBM cell proliferation by competitively binding miR-195 and negatively regulating the miR-195/CDC25A axis.

### Knocking down OXCT1-AS1 inhibits GBM growth in vivo

**Fig. 8:**
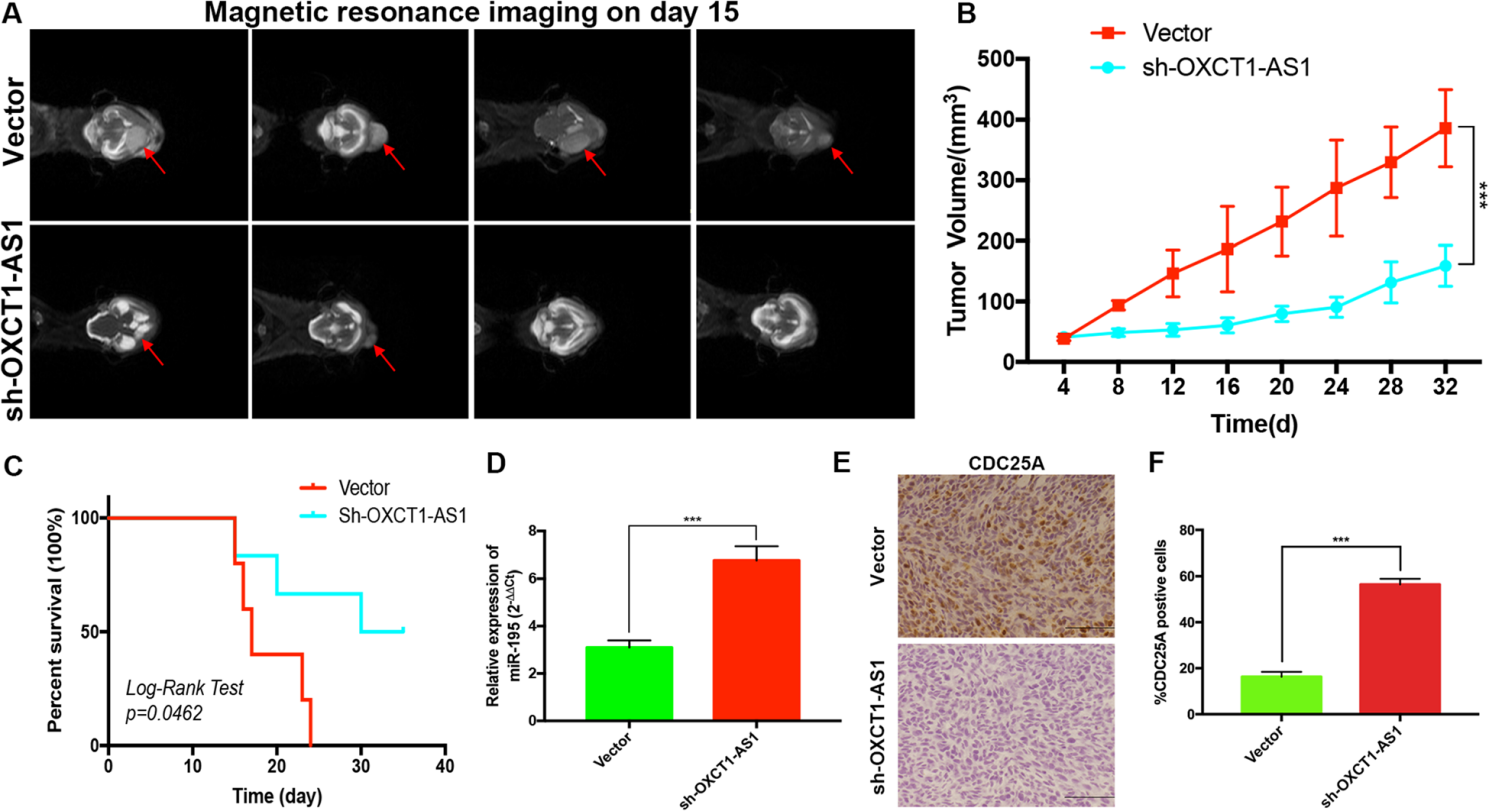
OXCT1-AS1 promotes the tumorigenesis of GBM cells in vivo. **a** After constructing intracranial tumour models (6 mice in each group), magnetic resonance imaging on day 15 showed that the tumour size in the sh-OXCT1-AS1 group was dramatically smaller than that in the vector group. **b** The tumour volume changes were calculated every 4 days. Tumour size = 4/3π(a*b*c), (a,b,c are the 1/2 maximum diameter at coronal, sagittal and axial position). **c** Survival status of nude mice bearing GBM. **d** Expression of miR-195 in nude mice was detected by qRT-PCR. **e-f** Immunohistochemistry of CDC25A in vivo, Scale bars: 150 nm. The bars represent SD (*p < 0.05, **p < 0.01, ***p < 0.001)

To investigate the impact of OXCT1-AS1 on tumorigenesis, an in vivo experiment was conducted. Primary GBM cell-derived mouse intracranial tumours were obviously smaller after knocking down OXCT1-AS1 compared with those derived from cells transfected with the empty vector (Fig. 8a and b). Consistently, the survival time in the OXCT1-AS1 silencing group was longer than that in the vector group (Fig. 8c). Next, qRT-PCR determined an obvious increase in miR-195 expression in the tumour tissue derived from sh-OXCT1-AS1-transfected cells (Fig. 8d). Through an immunohistochemistry assay, we found that the expression level of CDC25A was decreased in the OXCT1-AS1 blocking group compared with that in the vector group (Fig. 8e-f). These findings indicated that OXCT1-AS1 promotes GBM growth in vivo.

## Discussion

Because of the lack of effective therapeutic targets, patients with GBM usually have the worst survival rate [7, 27]. Thus, identifying new, suitable and effective therapeutic targets is critical [28]. In recent studies, deregulation of lncRNAs was revealed in various malignancies and implicated potential biomarkers and therapeutic targets [29–31]. For example, lncRNA LINC00899 suppresses breast cancer progression by inhibiting miR-425 [32], lncRNA ZFAS1 regulates oesophageal squamous cell carcinoma cell malignant behaviours via the miR-124/STAT3 axis [33] and lncRNA MALAT1 promotes GBM proliferation and progression by targeting the miR-199a/ZHX1 axis [34]. These studies indicated that lncRNAs play an important role in diagnosing and treating human cancers. In our present study, we identified DElncRNAs, DEmiRNAs and DEmRNAs in GBM and normal brain samples from the GEO database. We further constructed a putative ceRNA network and performed GO and KEGG pathway enrichment analyses. From the ceRNA network, we identified the core lncRNA OXCT1-AS1. In our subsequent studies, we focused on verifying that OXCT1-AS1 is a specific biomarker and potent therapeutic target for GBM. We identified that lncRNA OXCT1-AS1 was aberrantly expressed in GBM tissue and that increased OXCT1-AS1 expression was associated with poor median survival. This result was confirmed in an in vitro experiment, and blocking OXCT1-AS1 efficiently suppressed cell proliferation, induced cell cycle arrest and inhibited the migration and invasion and EMT related gene expression of GBM cells. It has been reported that ectopic miR-195 over-expression increases E-cadherin in prostate cancer and breast cancer cells [35, 36], these evidence may further support OXCT-AS1/miR-195 axis is associated with cell migration and invasion. In vivo experiments demonstrated that knocking down OXCT1-AS1 inhibited GBM growth. Collectively, our data demonstrated that OXCT1-AS1 acts as an oncogene in GBM and can become a specific diagnostic and prognostic biomarker as well as a therapeutic target in GBM.

To further investigate the potential mechanisms, we filtered the downregulated DEmiRNAs from the ceRNA network we constructed. Based on the ceRNA network hypothesis, we conducted bioinformatics analysis using LncBase V2.0 and showed that OXCT1-AS1 contained a conserved target site of miR-195. Previous studies have proven that miR-195 acts as a tumour suppressor in various cancers [37–39]. In our study, we explored the potential targets of miR-195. According to KEGG enrichment analysis, we screened the DEmRNAs matched with the cell cycle pathway and used TargetScan to predict conserved target sites. We found that CDC25A can be the direct target of miR-195. CDC25A is a member of the CDC25 phosphatase family and plays an important role in cell cycle regulation by activating cyclins-CDKs through the removal of inhibitory phosphates [40]. Ectopic CDC25A expression accelerates the G1/S phase transition [41, 42]. In our study, knocking down OXCT1-AS1 reduced the CDC25A expression level and increased the CDK2 phosphorylation level. Flow cytometry assays also confirmed that OXCT1-AS1 knockdown caused cell cycle arrest. Additionally, luciferase activity assays and functional experiments demonstrated that OXCT1-AS1 promotes GBM proliferation by acting as a ceRNA and regulating the miR-195/CDC25A axis.

All in all, OXCT1-AS1, a novel potential prognostic factor and therapeutic target, is closely related to the malignant proliferation of GBM. High expression of OXCT1-AS1 is associated with poor prognosis and survival rate, especially in patients with recurrence GBM (Fig. S2). How to apply our gene to clinical treatment is also the focus of our future research. Recent advancements in CRISPR/Cas9 technologies for gene knockout and point mutations may facilitate the development of OXCT1-AS1 targeted cancer therapy.

## Conclusions

In the present study, we first identified lncRNA OXCT1-AS1 as a potential predictor and therapeutic target of GBM. Knocking down OXCT1-AS1 significantly inhibited proliferation, migration, invasion and tumorigenesis in GBM. We also investigated the molecular mechanism involved and demonstrated that OXCT1-AS1 might act as a ceRNA and regulate the miR-195/CDC25A axis. Our findings reveal a novel regulatory network in GBM that can help gain insight into the pathogenesis of GBM and improve the treatment of GBM patients.

## Supplementary Information

**Additional file 1: Figure S1.** Heatmaps of DEmRNAs in the GSE4290 dataset (A), DEmiRNAs in the GSE90603 dataset (B) and DElncRNAs in the GSE104267 dataset.

**Additional file 2: Table S1.** GO term enrichment for biological processes of altered genes in the ceRNA network.

**Additional file 3: Table S2.** GO term enrichment for cellular components of altered genes in the ceRNA network.

**Additional file 4: Table S3.** GO term enrichment for molecular functions of altered genes in the ceRNA network.

**Additional file 5: Table S4.** Antibodies used in this study.

**Additional file 6: Table S5.** qRT-PCR primer sequences used in this study.

**Additional file 7: Figure S2.** OXCT1-AS1 is significantly associated with poor prognosis in patients with recurrent GBM. (A) OXCT1-AS1 expression patterns in patients with recurrent GBM and the survival time and status from the TCGA database. (B) Kaplan-Meier curve analysis of OXCT1-AS1 based on TCGA recurrent GBM samples. (C) ROC curve of OXCT1-AS1 and poor prognosis in patients with recurrent GBM.

## Data Availability

Because of our internal policy, raw data cannot be shared.

## Abbreviations

lncRNAs: Long noncoding RNAs
GBM: Glioblastoma
ceRNA: Competing endogenous RNA
OXCT1-AS1: OXCT1 antisense RNA 1
GEO: Gene Expression Omnibus
TCGA: The Cancer Genome Atlas
qRT-PCR: Quantitative real-time PCR
DEmRNAs: Differentially expressed mRNAs
DEmiRNAs: Differentially expressed miRNAs
DElncRNAs: Differentially expressed lncRNAs
GO: Gene Ontology
KEGG: Kyoto Encyclopedia of Genes and Genome
PPI: Protein-protein interaction
STRING: Search Tool for the Retrieval of Interacting Genes
CDC25A: Cell division cycle 25A
