# Supplementary figures and images for: Novel LncRNA OXCT1-AS1 indicates poor prognosis and contributes to tumorigenesis by regulating miR-195/CDC25A axis in glioblastoma
Source: J Exp Clin Cancer Res. 2021 Apr 8;40:123. doi: 10.1186/s13046-021-01928-4 (PMC8028723; doi:10.1186/s13046-021-01928-4)

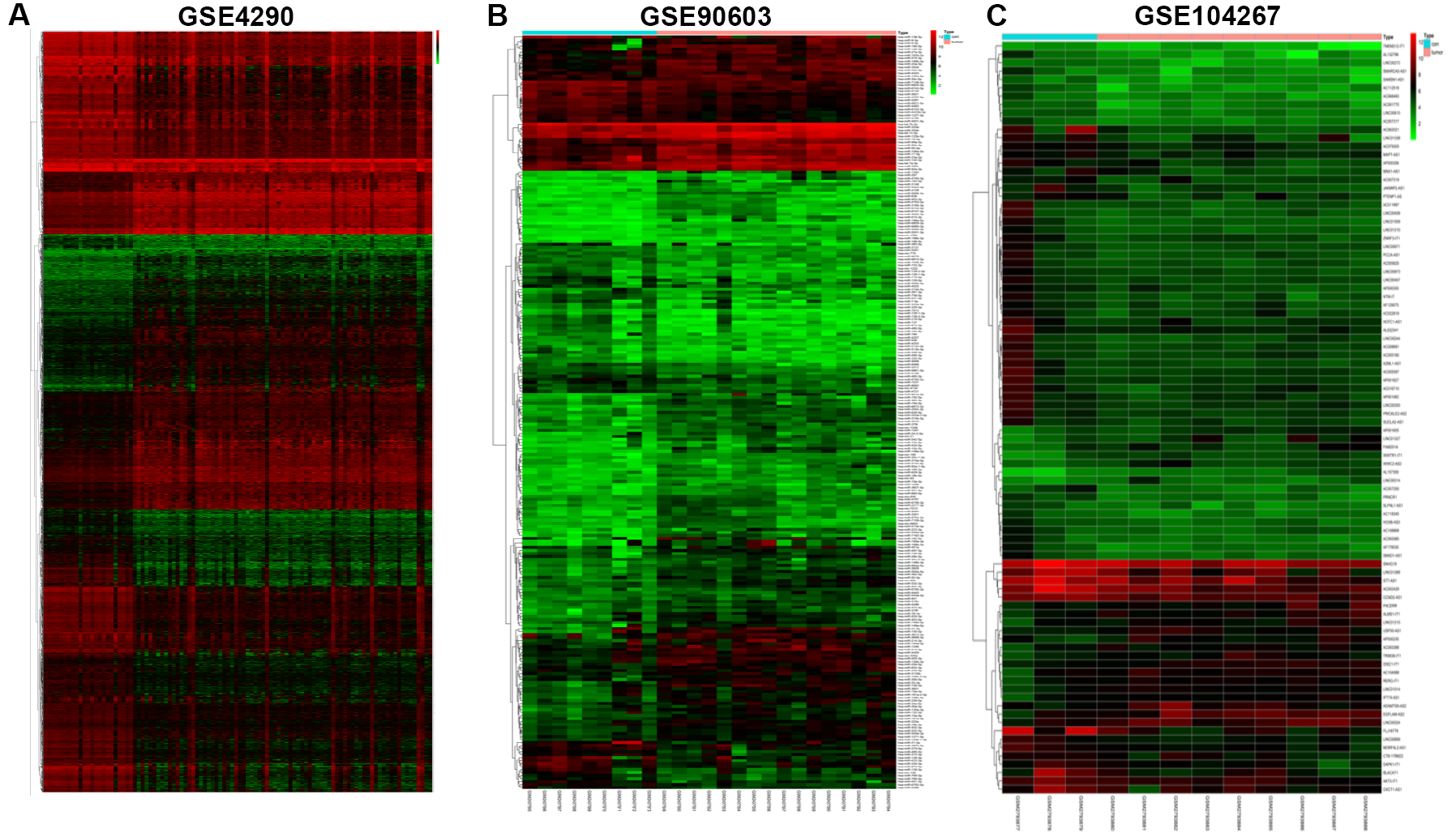

Supplement: Supplementary file 1 — Additional file 1: Figure S1. Heatmaps of DEmRNAs in the GSE4290 dataset (A), DEmiRNAs in the GSE90603 dataset (B) and DElncRNAs in the GSE104267 dataset. [file 13046_2021_1928_MOESM1_ESM.png]

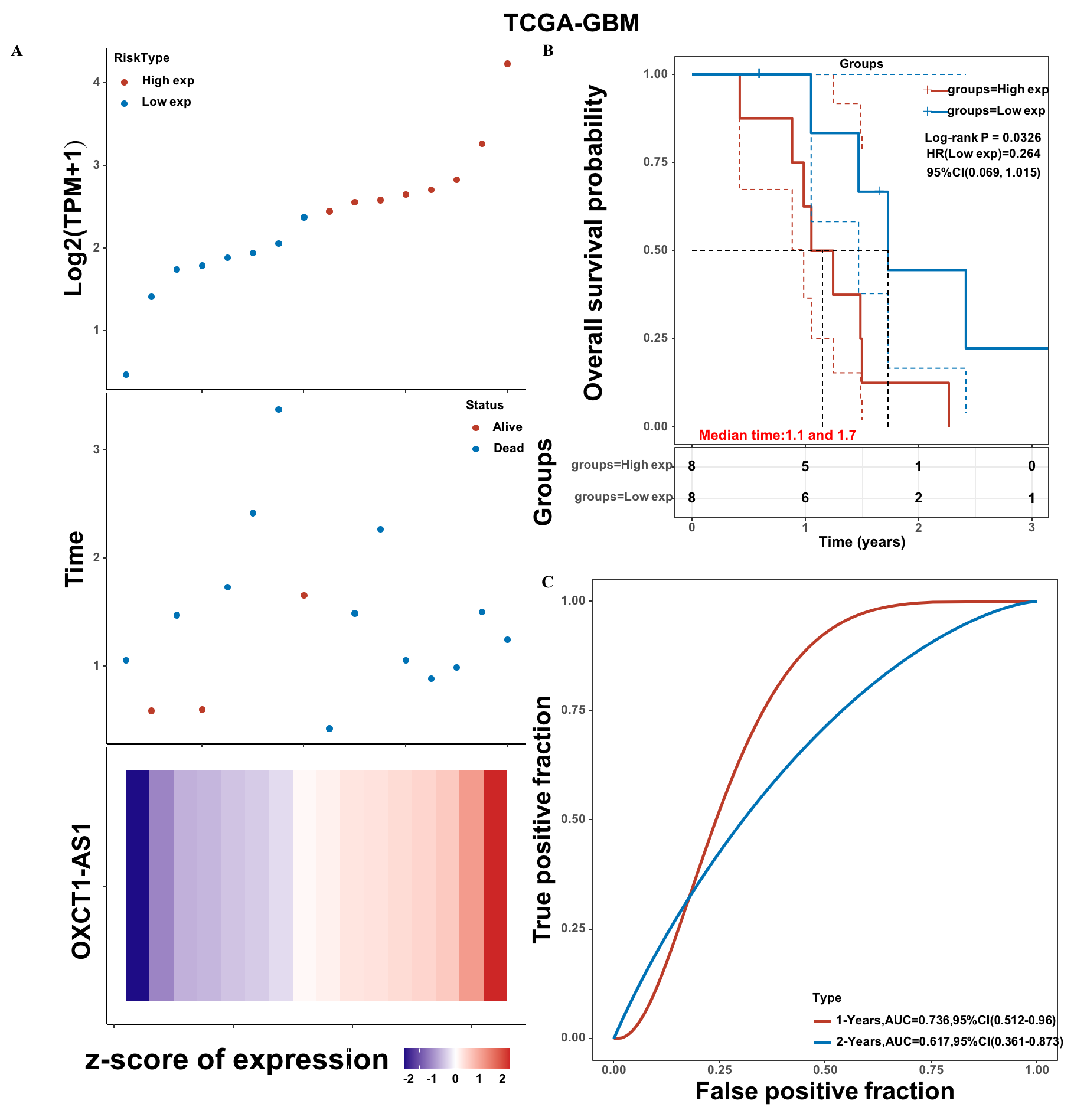

Supplement: Supplementary file 7 — Additional file 7: Figure S2. OXCT1-AS1 is significantly associated with poor prognosis in patients with recurrent GBM. (A) OXCT1-AS1 expression patterns in patients with recurrent GBM and the survival time and status from the TCGA database. (B) Kaplan-Meier curve analysis of OXCT1-AS1 based on TCGA recurrent GBM samples. (C) ROC curve of OXCT1-AS1 and poor prognosis in patients with recurrent GBM. [file 13046_2021_1928_MOESM7_ESM.png]
