# Supplementary material for: Novel LncRNA OXCT1-AS1 indicates poor prognosis and contributes to tumorigenesis by regulating miR-195/CDC25A axis in glioblastoma
Source: J Exp Clin Cancer Res. 2021 Apr 8;40:123. doi: 10.1186/s13046-021-01928-4 (PMC8028723; doi:10.1186/s13046-021-01928-4)
Supplement: Supplementary file 2 — Additional file 2: Table S1. GO term enrichment for biological processes of altered genes in the ceRNA network. [file 13046_2021_1928_MOESM2_ESM.docx]

| **ID** | **Description** | **Adjusted P values** | **Counts** | **Gene names** |
| --- | --- | --- | --- | --- |
| GO:0043065 | positive regulation of apoptosis | 0.00051 | 11 | TXNIP, NOTCH2, CDKN1A, EPHA7, MAP3K1, PPP3R1, STK17B, SOX4, MAPK9, TIMP3, TP53INP1 |
| GO:0043068 | positive regulation of programmed cell death | 0.00054 | 11 | TXNIP, NOTCH2, CDKN1A, EPHA7, MAP3K1, PPP3R1, STK17B, SOX4, MAPK9, TIMP3, TP53INP1 |
| GO:0010942 | positive regulation of cell death | 0.00056 | 11 | TXNIP, NOTCH2, CDKN1A, EPHA7, MAP3K1, PPP3R1, STK17B, SOX4, MAPK9, TIMP3, TP53INP1 |
| GO:0042981 | regulation of apoptosis | 0.00073 | 15 | MEF2C, TXNIP, NOTCH2, CDKN1A, EPHA7, MCL1, MAP3K1, VEGFA, STK17B, PPP3R1, MAPK9, SOX4, BCL2L2, TIMP3, TP53INP1 |
| GO:0043067 | regulation of programmed cell death | 0.00080 | 15 | MEF2C, TXNIP, NOTCH2, CDKN1A, EPHA7, MCL1, MAP3K1, VEGFA, STK17B, PPP3R1, MAPK9, SOX4, BCL2L2, TIMP3, TP53INP1 |
| GO:0010941 | regulation of cell death | 0.00083 | 15 | MEF2C, TXNIP, NOTCH2, CDKN1A, EPHA7, MCL1, MAP3K1, VEGFA, STK17B, PPP3R1, MAPK9, SOX4, BCL2L2, TIMP3, TP53INP1 |
| GO:0022402 | cell cycle process | 0.00116 | 12 | KIF23, NOTCH2, CDKN1A, BTRC, CYP26B1, CHEK1, CEP55, HAUS8, MAPRE3, CDC25A, WEE1, TP53INP1 |
| GO:0001709 | cell fate determination | 0.00130 | 4 | NOTCH2, MCL1, CYP26B1, JAG1 |
| GO:0007049 | cell cycle | 0.00162 | 14 | KIF23, TXNIP, E2F7, BTRC, CHEK1, CEP55, WEE1, CDC25A, NOTCH2, CDKN1A, CYP26B1, HAUS8, MAPRE3, TP53INP1 |
| GO:0000278 | mitotic cell cycle | 0.00298 | 9 | KIF23, CDKN1A, BTRC, CHEK1, CEP55, HAUS8, MAPRE3, CDC25A, WEE1 |
