# Supplementary material for: Novel LncRNA OXCT1-AS1 indicates poor prognosis and contributes to tumorigenesis by regulating miR-195/CDC25A axis in glioblastoma
Source: J Exp Clin Cancer Res. 2021 Apr 8;40:123. doi: 10.1186/s13046-021-01928-4 (PMC8028723; doi:10.1186/s13046-021-01928-4)
Supplement: Supplementary file 3 — Additional file 3: Table S2. GO term enrichment for cellular components of altered genes in the ceRNA network. [file 13046_2021_1928_MOESM3_ESM.docx]

| **ID** | **Description** | **Adjusted P values** | **Counts** | **Gene names** |
| --- | --- | --- | --- | --- |
| GO:0031974 | membrane enclosed lumen | 0.000903 | 24 | MEF2C, KIF23, TXNIP, STK38, MCL1, E2F5, E2F7, SMAD5, PDIA6, CHEK1, CERCAM, CBFA2T3, ITPR1, CDC25A, WEE1, CALU, CDKN1A, HIF1A, OSR1, VEGFA, HOXA10, MKX, PBX3, OXR1 |
| GO:0005654 | nucleoplasm | 0.001187 | 15 | KIF23, MEF2C, STK38, E2F5, MCL1, E2F7, SMAD5, CHEK1, CBFA2T3, WEE1, CDC25A, CDKN1A, HIF1A, HOXA10, PBX3 |
| GO:0044432 | endoplasmic reticulum part | 0.001561 | 9 | ARHGAP32, SLC9A6, CYP26B1, PDIA6, CERCAM, JPH1, TRAM1, ITPR1, CALU |
| GO:0043233 | organelle lumen | 0.001680 | 23 | MEF2C, KIF23, STK38, E2F5, MCL1, E2F7, SMAD5, PDIA6, CHEK1, CERCAM, CBFA2T3, ITPR1, CDC25A, WEE1, CALU, CDKN1A, HIF1A, OSR1, VEGFA, HOXA10, MKX, PBX3, OXR1 |
| GO:0044430 | cytoskeletal part | 0.002438 | 15 | KIF23, MYO5A, LIMA1, GABARAPL1, SH3PXD2A, CALD1, TANC1, CHEK1, CEP55, TPM2, ITPR1, ARHGAP32, NDEL1, HAUS8, MAPRE3 |
| GO:0070013 | intracellular organelle lumen | 0.002956 | 22 | MEF2C, KIF23, STK38, E2F5, MCL1, E2F7, SMAD5, PDIA6, CHEK1, CERCAM, CBFA2T3, ITPR1, CDC25A, WEE1, CALU, CDKN1A, HIF1A, OSR1, HOXA10, MKX, PBX3, OXR1 |
| GO:0031981 | nuclear lumen | 0.003677 | 19 | MEF2C, KIF23, STK38, E2F5, MCL1, E2F7, SMAD5, CHEK1, CBFA2T3, ITPR1, WEE1, CDC25A, CDKN1A, HIF1A, OSR1, HOXA10, MKX, PBX3, OXR1 |
| GO:0019898 | extrinsic to membrane | 0.003945 | 10 | RAB11FIP2, NAPG, RASGRP1, CYP26B1, GNB5, BCL2L2, RAPGEF4, PXK, JPH1, PLEKHA1 |
| GO:0005783 | endoplasmic reticulum | 0.007034 | 14 | ARHGAP32, GABARAPL1, SLC9A6, NAPG, RASGRP1, CYP26B1, PDIA6, ELOVL7, PTGFRN, CERCAM, JPH1, TRAM1, ITPR1, CALU |
| GO:0015629 | actin cytoskeleton | 0.007142 | 7 | MYO5A, ARHGAP32, LIMA1, SH3PXD2A, CALD1, STK17B, TPM2 |
