# Supplementary material for: Novel LncRNA OXCT1-AS1 indicates poor prognosis and contributes to tumorigenesis by regulating miR-195/CDC25A axis in glioblastoma
Source: J Exp Clin Cancer Res. 2021 Apr 8;40:123. doi: 10.1186/s13046-021-01928-4 (PMC8028723; doi:10.1186/s13046-021-01928-4)
Supplement: Supplementary file 4 — Additional file 4: Table S3. GO term enrichment for molecular functions of altered genes in the ceRNA network. [file 13046_2021_1928_MOESM4_ESM.docx]

| **ID** | **Description** | **Adjusted P values** | **Counts** | **Gene names** |
| --- | --- | --- | --- | --- |
| GO:0003700 | transcription factor activity | 0.00118238 | 16 | MEF2C, SATB1, E2F5, E2F7, SMAD5, RUNX1T1, ATAD2, SOX4, ZNF367, CBFA2T3, ZNF217, HOXA3, HIF1A, HOXA10, MKX, PBX3 |
| GO:0008092 | cytoskeletal protein binding | 0.0047829 | 10 | MYO5A, GABARAPL1, LIMA1, YWHAH, NDEL1, CALD1, PXK, SSX2IP, TPM2, MAPRE3 |
| GO:0005543 | phospholipid binding | 0.00548634 | 6 | ARHGAP32, SH3PXD2A, PXK, ABCA1, ITPR1, PLEKHA1 |
| GO:0030528 | transcription regulator activity | 0.00636018 | 19 | MEF2C, SATB1, E2F5, E2F7, SMAD5, RUNX1T1, ATAD2, SOX4, ZNF367, CBFA2T3, NOTCH2, YWHAH, ZNF217, HOXA3, HIF1A, NCOA3, HOXA10, MKX, PBX3 |
| GO:0008289 | lipid binding | 0.00796319 | 9 | ARHGAP32, SH3PXD2A, MAP3K1, RASGRP1, CYP26B1, PXK, ABCA1, ITPR1, PLEKHA1 |
| GO:0035091 | phosphoinositide binding | 0.02614161 | 4 | ARHGAP32, SH3PXD2A, PXK, ITPR1 |
| GO:0051019 | mitogen-activated protein kinase binding | 0.03156358 | 2 | DUSP2, MAP3K1 |
| GO:0003779 | actin binding | 0.05757975 | 6 | MYO5A, LIMA1, YWHAH, CALD1, PXK, TPM2 |
| GO:0004721 | phosphoprotein phosphatase activity | 0.08888871 | 4 | PTPRD, DUSP2, PPP3R1, CDC25A |
| GO:0004672 | protein kinase activity | 0.09232516 | 8 | EPHA7, STK38, MAP3K1, STK17B, MAPK9, CHEK1, PXK, WEE1 |
