# Supplementary material for: Novel LncRNA OXCT1-AS1 indicates poor prognosis and contributes to tumorigenesis by regulating miR-195/CDC25A axis in glioblastoma
Source: J Exp Clin Cancer Res. 2021 Apr 8;40:123. doi: 10.1186/s13046-021-01928-4 (PMC8028723; doi:10.1186/s13046-021-01928-4)
Supplement: Supplementary file 5 — Additional file 5: Table S4. Antibodies used in this study. [file 13046_2021_1928_MOESM5_ESM.docx]

| **Antibody** | **Vendor** | **Catalogue number** | **Technique** |
| --- | --- | --- | --- |
| KI67 | Abcam | Ab15580 | IF |
| CDC25A | Affinity Biosciences | AF6252 | western blot、IHC |
| CCNA1 | Affinity Biosciences | AF0143 | western blot |
| CCNE1 | Affinity Biosciences | AF0144 | western blot |
| p-CDK2 | Affinity Biosciences | AF3237 | western blot |
| CDK2 | Affinity Biosciences | AF6237 | western blot |
| β-Actin | Cell Signaling Technology | #3700 | western blot |
