# Supplementary material for: Novel LncRNA OXCT1-AS1 indicates poor prognosis and contributes to tumorigenesis by regulating miR-195/CDC25A axis in glioblastoma
Source: J Exp Clin Cancer Res. 2021 Apr 8;40:123. doi: 10.1186/s13046-021-01928-4 (PMC8028723; doi:10.1186/s13046-021-01928-4)
Supplement: Supplementary file 6 — Additional file 6: Table S5. qRT-PCR primer sequences used in this study. [file 13046_2021_1928_MOESM6_ESM.docx]

| **Gene name** | **Primer forward sequence** | **Primer reverse sequence** |
| --- | --- | --- |
| OXCT1-AS1 | CCTGGACTGCGTTCACGTTT | CTGCAAGCCTTGTTGCTCAC |
| CDC25A | CACTGGAGGTGAAGAACAACAG | CAGCCACGAGATACAGGTCTTA |
| GAPDH | ACCACAGTCCATGCCATCAC | TCCACCACCCTGTTGCTGTA |
| miR-195 | CGCAGCACAGAAATATTGGC | CTCAACTGGTGTCGTGGAGTC |
| U6 | CTCGCTTCGGCAGCACA | AACGCTTCACGAATTTGCGT |
|  | **Reverse-transcribed primer** | |
| miR-195 | CTCAACTGGTGTCGTGGAGTCGGCAATTCAGTTGAGGCCAATAT | |
| U6 | AACGCTTCACGAATTTGCGT | |
